# Psychopathological symptoms and work status of Southeastern Brazilian nursing in the context of COVID-19

**DOI:** 10.1590/1518-8345.5768.3518

**Published:** 2022-03-21

**Authors:** Jheynny Sousa Alves, Angelica Martins de Souza Gonçalves, Marina Nolli Bittencourt, Verônica de Medeiros Alves, Darcio Tadeu Mendes, Maria do Perpétuo Socorro de Sousa Nóbrega

**Affiliations:** 1 Universidade Federal de São Carlos, São Carlos, SP, Brasil.; 2 Universidade Federal de São Carlos, Grupo de Pesquisa Saúde Mental no Contexto da Reforma Psiquiátrica, São Carlos, SP, Brasil.; 3 Bolsista da Coordenação de Aperfeiçoamento de Pessoal de Nível Superior (CAPES), Brasil.; 4 Universidade Federal de São Carlos, Departamento de Enfermagem, São Carlos, SP, Brasil.; 5 Universidade Federal de Mato Grosso, Faculdade de Enfermagem, Cuiabá, MT, Brasil.; 6 Universidade Federal de Alagoas, Escola de Enfermagem, Maceió, AL, Brasil.; 7 Universidade de São Paulo, Escola de Enfermagem, Departamento de Enfermagem Materno-Infantil e Psiquiátrica, Núcleo de Estudos e Pesquisas em Enfermagem em Adições e Saúde Mental (NEPEASM), São Paulo, SP, Brasil.; 8 Universidade de São Paulo, Escola de Enfermagem, Departamento de Enfermagem Materno-Infantil e Psiquiátrica, Núcleo de Estudos e Pesquisas em Enfermagem em Adições e Saúde Mental (NEPEASM), São Paulo, SP, Brasil.

**Keywords:** Nurse Practitioners, COVID-19, Mental Health, Psychopathology, Mental Disorders, Pandemics, Profissionais de Enfermagem, COVID-19, Saúde Mental, Psicopatologia, Transtornos Mentais, Pandemias, Enfermeras Practicantes, COVID-19, Salud Mental, Psicopatología, Trastornos Mentales, Pandemias

## Abstract

**Objective:**

to evaluate the relationship between psychopathological symptoms and the work situation of nursing professionals in the Southeast Region, Brazil, in the context of the COVID-19 pandemic.

**Method:**

an observational and cross-sectional study with virtual and snowball data collection from April to July 2020. A questionnaire containing socio-demographic and labor data and the psychopathological symptoms assessment scale (psychoticism, obsessiveness/compulsivity, somatization, and anxiety) were applied. Descriptive and inferential statistics were used to analyze the data.

**Results:**

among the 532 participants, there was a relationship between weekly workload and psychoticism. All domains of the scale were associated with embarrassment and/or violence in the course of work and receiving psychological/emotional support from the institution where the individual works/studies.

**Conclusion:**

the age group, heavy workload, experienced violence and lack of psychological support during the pandemic were associated with increased psychopathological symptoms among nursing professionals. It is suggested the creation of institutional guidelines aimed at the reception and follow-up of these demands.

## Resumo

**Objetivo:**

avaliar a relação entre sintomas psicopatológicos e a situação laboral de profissionais de enfermagem da Região Sudeste do Brasil, no contexto da pandemia da COVID-19.

**Método:**

estudo observacional e transversal com coleta de dados virtual e por meio de *snowball* nos meses de abril a julho de 2020. Aplicou-se um questionário contendo dados sociodemográficos, laborais e a escala de avaliação de sintomas psicopatológicos (psicoticismo, obsessividade/compulsividade, somatização e ansiedade). Foram utilizadas estatísticas descritivas e inferenciais para a análise dos dados.

**Resultados:**

entre os 532 participantes, verificou-se relação entre carga horária de trabalho semanal e psicoticismo. Todos os domínios da escala associaram-se ao constrangimento e/ou violência no percurso de trabalho e recebimento de suporte psicológico/emocional da instituição onde o indivíduo trabalha/estuda.

**Conclusão:**

a faixa etária, carga horária elevada, violência vivenciada e falta de suporte psicológico durante a pandemia associaram-se com o aumento dos sintomas psicopatológicos entre os profissionais de enfermagem. Sugere-se a criação de diretrizes institucionais voltadas ao acolhimento e acompanhamento dessas demandas.

## Resumen

**Objetivo:**

evaluar la relación entre síntomas psicopatológicos y la situación laboral de profesionales de enfermería de la Región Sureste de Brasil, en el contexto de la pandemia de la COVID-19.

**Método:**

estudio observacional y transversal con recolección de datos virtual y por medio de *snowball* en los meses de abril a julio de 2020. Se aplicó un cuestionario que contenía datos sociodemográficos, laborales y la escala de evaluación de síntomas psicopatológicos (psicoticismo, obsesión/compulsión, somatización y ansiedad). Fueron utilizadas estadísticas descriptivas y de inferencia para el análisis de los datos.

**Resultados:**

entre los 532 participantes, se verificó una relación entre la carga horaria de trabajo semanal y el psicoticismo. Todos los dominios de la escala se asociaron con incomodidad y/o violencia, en el transcurso del trabajo y el recibimiento de soporte psicológico/emocional de la institución, en donde el individuo trabaja/estudia.

**Conclusión:**

el intervalo etario, la carga horaria elevada, la violencia experimentada y la falta de soporte psicológico, durante la pandemia se asociaron con el aumento de los síntomas psicopatológicos entre los profesionales de enfermería. Se sugiere la creación de directrices institucionales dirigidas al acogimiento y acompañamiento de esas demandas.

**Highlights**

(1) Significant results between psychopathological symptoms and work factors.

(2) Workload of the nursing staff was related to psychoticism.

(3) All investigated mental symptoms were related to age and embarrassment.

(4) Sex was associated with psychoticism and obsessiveness/compulsivity.

## Introduction

The rapid transmission of the SARS-COV-2 virus from its dissemination in large urban centers, the high attack rates (ratio of total cases to total exposed) and mortality from COVID-19 in the various regions of Brazil^1^ ^-^ ^2^ required adaptation of the public and private systems, as well as of the human resources in health, to meet the emergency demands^3^. In São Paulo State the first cases of COVID-19 were confirmed in February 2020^1^ ^-^ ^2^. Thus, from then on, a new work routine was established for health professionals.

Evidence shows that health care professionals, namely nurses, working on the frontline of care for people with COVID-19, were exposed to a high risk of infection by the virus and presented severe degrees of psychological distress^4^. In Brazil, mapping of the index of this risk at the beginning of the pandemic showed the alarming figure of 97 to 100% of infection^5^. From the labor point of view, the country accounts for an expressive total number of deaths among nursing professionals, with the Southeast Region having the highest number (n=838, in July 2021), and the State of São Paulo, the most lethal^6^. 

The new work reality imposed probably accentuated, even more, the significant risks of physical and mental illness already inherent in the performance of nursing work in the national reality^7^ ^-^ ^8^. In relation to mental health, work in the context of COVID-19 was found to have the potential to affect health workers’ attention, understanding and decision-making capacity and to generate lasting impacts on their overall well-being^9^. Specifically, pictures of depression, anxiety and stress have increased, not only in the general population, but especially among health professionals^4^.

Measures to mitigate the impacts of mental suffering due to the pandemic of COVID-19 cannot be neglected^10^ ^-^ ^11^, since previous experiences have shown that these can last longer and be more prevalent than the epidemic itself, thus having incalculable psychosocial ramifications^12^ ^-^ ^13^.

Given the above and the gap regarding the tracking of psychopathological symptoms presented by nursing professionals working in the most populated and technology-dense region of Brazil, investigating aspects of mental health may be useful to generate metrics regarding mental disorders that emerged in the work context of the largest force of healthcare workers during the COVID-19 pandemic. Thus, this research aimed to evaluate the relationship between psychopathological symptoms and the work situation of nursing professionals in the Southeast Region of Brazil in the context of the COVID-19 pandemic.

## Method

### Study design

Observational and cross-sectional study, guided by the tool STROBE (Strengthening the Reporting of Observational Studies in Epidemiology)^14^.

### Sample

Non-probabilistic sample, using the snowball technique, composed of nursing professionals (nurses, technicians, nursing assistants and midwives) from the Southeast Region of Brazil who met the following eligibility criteria: exercising activities at any level of health care, regardless of the activity (direct assistance and/or administrative/managerial) or who worked in teaching and research during the COVID-19 pandemic, residents of the Southeast Region of Brazil. A heterogeneous sample was chosen in order to show the predisposition to secondary traumatization of nursing professionals due to their identification with the suffering of their peers in front-line work^12^ and, also, because in March 2020 the Strategic Action “Brazil counts on me^15^ and with it many nursing education workers have been supervising care in order to expand the coverage of care to the population at all levels of care.

### Data collection

The data was collected, virtually, in the period from April to July 2020 through Google® Questionnaires, made available on social networks through a link with an invitation to participate in the survey, containing: a) Free and Informed Consent Term (FICT); b) sociodemographic and labor information (age, sex, race, occupation, marital status, nationality, state of residence, income, professional performance, nature of the work institution, time of training, time of performance, work situation, performance as a nursing professional at the moment, weekly workload, direct performance in assistance, service of performance, level of satisfaction in the development of work activities, suffering due to constraints and/or violence during the course of work since the beginning of the pandemic, nature of COVID-19 cases in the workplace/study, nature of patients in relation to COVID-19 in the workplace/study, receiving psychological/emotional support/support from the workplace/study institution in the context of COVID-19, and type of support received); c) Symptom Assessment Scale-40 (SAS-40), derived from the Symptom Checklist-90-R (SCL-90-R)^16^ which was adapted and validated for Brazil in 2001^17^. In this study, we used the 40-item version (SAS-40); it is a self-report screening scale for psychopathological symptoms based on the last fourteen days. It was validated for the non-clinical population in Brazil and consists of four subscales, with 10 items in each: 1. psychoticism: evaluates psychosis, depression, hostility symptoms, and paranoid ideas; 2. obsessiveness/compulsiveness: assesses symptoms of repeated thoughts and actions, accompanied by discomfort in interpersonal relationships; 3. Somatization: comprises symptoms common to somatic and somatoform disorders and 4. Anxiety: comprises symptoms of generalized anxiety, phobic anxiety related to objects or situations. The SAS-40 is self-administered and the response pattern consists of a Likert-type scale with three levels of intensity: 0 = no symptoms; 1 = few symptoms; and 2 = many symptoms. The raw score is calculated by adding the values from 0 to 2 for each item answered in each dimension and divided by the number of items in each dimension. If the respondent does not answer any item on the scale, the division is made on the number of items answered^18^.

### Data treatment and analysis

The information was recorded in Excel spreadsheets and after double-checking, exported to the statistical program SPSS, version 22, for statistical analysis. The sample was characterized by descriptive statistics, using measures of central tendency (mean, mode, medians) and measures of dispersion (minimum, maximum, and standard deviations). To verify data normality, the Kolmogorov-Smirnov test was used; for the analysis of binary variables, Mann Whitney’s U test and for the analysis of multiple variables, the Kruskal-Wallis (KW) test was used. The confidence interval was 95% and the significance level adopted was 5% for all tests (p ≤ 0.05).

### Ethical aspects

The research met the standards of Resolution no:466/2012, of the National Health Council, approved by the National Research Ethics Committee (opinion no. 3,954,557 and CAAE: 30359220.4.0000.0008 of 2020). The FICT was entered into Google® Questionnaires so that the participant could accept or refuse/stop participation before accessing the questions. The participant proceeded to the step referring to the collection instruments when he selected the acceptance option in the FICT and inserted an e-mail for sending a copy. The participant was given the option to print the FICT form if he/she wished to do so. All participants accepted the FICT prior to responding to the data collection instruments.

## Results

A total of 532 nursing professionals between 20 and 87 years of age participated in the study (mean age of 37 years, with standard deviation of ±10.72). Of these, 474 (89.1%) were female, most lived with a partner, being 210 (39.5%) married and 70 (13.2%) in a stable union. Singles totaled 211 (39.7%), 39 (7.3%) were divorced or separated and 2 were widowed (0.4%). The Catholic religion was predominant with 186 (35.7%) of the participants. Table 1 shows the socio-demographic, clinical and pandemic context characterization of the nursing professionals who participated in the study.

**Table 1:** Profile of nursing professionals (n=532) from the Southeast Region. Brazil, 2020

Age group (years)	N(%)
20-39	342(64.3)
40-59	171(32.1)
≥60	19(3.6)
**Sex**	
Male	58(10.9)
Female	474(89.1)
**Current marital status**	
With partner	280 (52.6)
Without partner	252(47.4)
**Race**	
White	338(63.5)
Black	53(10.0)
Yellow	13(2.4)
Brown	126(23.7)
Indigenous	1(0.2)
None of the above	1(0.2)
**Nationality**	
Brazilian	526(98.9)
Foreigner	6(1.1)
Origin (States)	
São Paulo	450(84.6)
Minas Gerais	41(7.7)
Rio de Janeiro	37(7.0)
Espirito Santo	4(0.8)
Income (MW^*^)	
Less than one	17(3.2)
1 to 3	197(37.0)
4 to 6	193(36.3)
7 to 9	84(15.8)
Above 10	41(7.7)
**You have pre-existing disease(s)**	
Yes	166(31.2)
No	366(68.8)
**If yes, are you treated for this (these) pre-existing disease(s)^†^**	
Yes	146(88.0)
No	20(12.0)
**Did you have any kind of psychological support BEFORE the pandemic COVID19**	
Yes	148(27.8)
No	384(72.2)
**Had any kind of psychiatric treatment BEFORE the pandemic COVID19**	
Yes	139(26.1)
No	393(73.9)
**Did you use any psychiatric medication without a doctor’s prescription BEFORE the pandemic COVID19**	
Yes	69(13.0)
No	463(87.0)
**Primary responsibility to the family**	
Main caregiver	130(24.4)
Indirect caregiver	92(17.3)
Financial rovider	173(32.5)
Not aplicable	137(25.8)
**Family member, friend, neighbor, work/study colleague (post-graduate cases) infected by the COVID-19 virus**	
Yes	385(72.4)
No	122(22.9)
Do not know	25(4.7)
**Death of family member, friend, neighbor, work/study colleague due to COVID-19**	
Yes	137(25.8)
No	376(70.7)
Do not know	19(3.6)

* MW = Brazilian minimum wage, approximately 200 dollars. ^†^Refers only to n=166 who responded affirmatively on the question of having pre-existing diseases

Regarding the characterization of the professional profile of the study participants, the mean time of training was 16.31 years (standard deviation ±87.25) and the mean time working was 11.59 (standard deviation ±9.35). Regarding work activity, 56% are nurses, followed by nursing technicians (14.7%). Most, 54.6%, work in public institutions and receive between 1 and 3 minimum wages. It was observed the average time of training of 16.31 years and the average time of work of 11.59 years. 

The majority of the participants fell into the category of formal worker with a weekly workload of 40 hours (181-34%). Of these, 75.4% reported not having suffered any kind of embarrassment and/or violence in the course of their work. At that time, 338 (63.5%) were working in direct assistance (Table 2).

**Table 2:** Labor profile of nursing professionals (n=532) from the Southeast Region. Brazil, 2020

Work situation	N (%)
Salaried employee with a signed contract	270(50.8)
Salaried employee without a signed contract	36(6.8)
Public Servant	153(28.8)
Others	73(13.6)
**Workload (hours/week)**	
20	20(3.8)
36	125(23.5)
40	181(34.0)
44	51(9.6)
More than 44	98(18.4)
Leave of absence or vacation	57(10.7)
**At that time, I was working in direct assistance**	
Yes	338(63.5)
No, dismissed for suspected coronavirus infection	16(3.0)
No, I am off work due to a diagnosis of COVID-19	12(2.3)
No, I am on medical leave for other health reasons	9(1.7)
No, I am on vacation/leave of absence after returning from an international trip	20(3.8)
I do not work in direct assistance	137(25.7)

Table 3 presents the results referring to the psychopathological symptoms of Psychoticism and Obsessiveness/Compulsivity assessed by the SCL-40-R scores. There was a significant association between both domains and the socio-occupational profile of nursing professionals. As for Psychoticism, the association was between the age group; suffering embarrassment and/or violence in the course of their work since the beginning of the pandemic (in Table 3, “embarrassments and/or violence”); receiving psychological/emotional support from the institution where they work/study in the context of the pandemic (“Support/support” in Table 3) and the weekly workload. As for Obsessiveness/Compulsivity, an association was found between suffering embarrassment and/or violence in the course of work since the beginning of the pandemic and receiving psychological/emotional support from the institution in which you work/study in the context of the COVID-19 pandemic.

**Table 3:** Relationship between symptoms of psychoticism and obsessiveness/compulsiveness and work situation of nursing professionals (n=532) from the Southeast Region. Brazil, 2020

	Psychoticism		Obsessiveness/compulsivity	
	Mean±Sd	P-value	Mean±Sd	P-value
**Age group (years)**		<0.001*		0.016*
20-39	1.69±0.47		1.72±0.48	
40-59	1.57±0.49		1.65±0.51	
≥60	1.39±0.47		1.46±0.49	
**Sex**		0.039†		0.037†
Male	1.54±0.52		1.57±0.52	
Female	1.66±0.48		1.70±0.49	
**Workload (hours/week)**		0.035*		0.155*
20	1.57±0.39		1.57±0.35	
36	1.69±0.46		1.73±0.51	
40	1.61±0.49		1.63±0.47	
44	1.52±0.47		1.67±0.48	
Mais de 44	1.73±0.47		1.77±0.49	
**Constraints and/or violence**		<0.001*		<0.001*
Yes	1.84±0.46		1.88±0.51	
No	1.59±0.48		1.63±0.47	
**Support/backup**		0.003^†^		0.00†
Yes	1.54±0.45		1.58±0.49	
No	1.68±0.49		1.72±0.49	

* Kruskal-Wallis Test; ^†^Mann-Whitney U-test

Table 4 presents the results regarding the psychopathological symptoms of Somatization and Anxiety assessed by the domains of the SAS-40 and the work profile of nursing professionals. Somatization showed significant association to age group; suffering from constraints and/or violence in the course of work since the beginning of the pandemic (“Constraints and/or violence” in Table 4) and receiving psychological/emotional support or support by the institution where they work/study in the context of COVID-19 (“Support/support” in Table 4). Regarding Anxiety, it was also found an association with suffering embarrassment and/or violence in the course of work since the beginning of the pandemic and receiving psychological/emotional support by the institution where he/she works/studies in the context of COVID-19.

**Table 4:** Comparison analysis between somatization/anxiety and employment status of nursing professionals (n=532) from the Southeast Region. Brazil, 2020

	Somatization		Anxiety	
	Mean±Sd	P-value	Mean±Sd	P-value
**Age group (years)**		<0.001*		0.030*
20-39	1.73±0.53		1.46±0.46	
40-59	1.62±0.53		1.41±0.47	
≥60	1.35±0.48		1.28±0.48	
**Sex**		0.064†		0.232†
Male	1.59±0.59		1.41±0.53	
Female	1.69±0.52		1.44±0.46	
**Workload (hours/week)**		0.229*		0.180*
20	1.55±0.43		1.31±0.33	
36	1.73±0.53		1.45±0.45	
40	1.65±0.53		1.39±0.44	
44	1.62±0.51		1.45±0.48	
More than 44	1.75±0.54		1.53±0.51	
**Constraints and/or violence**		<0.001*		0.002*
Sim	1.84±0.51		1.56±0.51	
No	1.63±0.52		1.40±0.44	
**Workload (weekly hours)**		0.229*		0.180*
20	1.55±0.43		1.31±0.33	
**Support/backup**		0.006†		0.004†
Yes	1.59±0.53		1.36±0.45	
No	1.72±0.53		1.46±0.47	

* Kruskal-Wallis Test; ^†^Mann-Whitney U-test

## Discussion

As for the relationship between psychopathological symptoms and employment status, an association was found between age group and all domains of the SAS-40 instrument. This association, in the context of the pandemic introduced by COVID-19, can be attributed to the relationship between age over 60 years, considered as a higher risk of infection; a retrospective and comparative study between young and middle-aged/elderly Chinese patients with COVID-19 found that the older population is more susceptible to the disease and is more likely to be admitted to intensive care and with a higher mortality rate^19^. In this perspective, we point out the alarming factor that involves the nursing professional whose work with patients infected by the virus is added to the fact of being more prone to the comorbidities presented by the disease, if they are over 60 years old, which contributes to worsen the situation. 

Clinical outcomes and length of hospitalization correlated directly with the underlying conditions and age of the COVID-19 patient. Similar findings to these were found in a study in China of 633 COVID-19 patients, which concluded that those older than 60 years are more likely to exhibit a more severe form of the disease, as during the study, 25 patients with a median age of 69.3 years died, inferring an effective mortality rate of 3.77%^20^.

Still regarding the age group, another Chinese study conducted with 606 health professionals with a mean age of 35.7 years and using the SCL-90-R scale to investigate the emergence of psychopathological symptoms in the context of the COVID-19 pandemic identified that the percentage of anxiety, somatic, and insomnia symptoms was 45.4%, 12.0%, and 32%, respectively. The frequency of somatic symptoms among participants with anxiety symptoms was 22.9%. Unlike the present study, no differences were found regarding socio-demographic and labor variables between participants with and without somatic symptoms^21^.

A study prior to the pandemic of COVID-19, however, conducted in the context of the acute respiratory syndrome outbreak in China showed that the occurrence of psychiatric symptoms among nurses was related to younger age and poor family support^22^. 

As for gender, there was an association with the Psychoticism and Obsessiveness/Compulsiveness domains. A cross-sectional study conducted in China with 1,257 health professionals distributed in 34 hospitals equipped to care for patients with COVID-19 found a considerable portion of these professionals with symptoms of depression, anxiety, insomnia, and distress. Women, nurses, and people living in Wuhan, as well as healthcare workers involved in the diagnosis, treatment, or nursing care of patients with suspected or confirmed COVID-19, were the group most affected by symptoms^23^. 

The weekly workload was associated with the Psychoticism domain. Regarding weekly workload, most participants 181 (34%) work 40 hours *per* week. With the pandemic introduced by COVID-19, the country’s health system was impacted, among other challenges, by the lack of health professionals and the need for increased workload, to perform patient care^24^ ^-^ ^25^. 

Another study conducted with health professionals in China shows that they had long working hours, performed several consecutive shifts, facts that had a direct impact on physical and mental fatigue^9^. Thus, it is important to emphasize that the health situation increased both mental stress and physical fatigue conditions, since the professionals carried out their activities in situations of overload of functions, extensive workload, risky situations, inadequate physical structure, scarcity of material resources and lack of professional training^26^. It is also worth remembering the importance of social support and collective coping strategies as a protective factor for workers exposed to intense work overload^27^. 

Chinese research that used the SCL-90-R and compared psychopathological symptoms between medical and non-medical professionals found that physicians had a higher prevalence of insomnia, anxiety, depression, somatization, and obsessive-compulsive symptoms^28^. Although the present research did not focus on prevalence, the results also showed the presence of psychoticism, obsessive-compulsive disorder, somatization and anxiety among nursing professionals in Southeastern Brazil. 

The variable suffering from constraints and/or violence in the course of work since the beginning of the pandemic COVID-19 was associated with all domains of the scale used in this study, which is derived from the aforementioned one. Health professionals represent a quarter of all cases of violence perpetrated at work, of these, nursing workers were the most affected^29^ ^-^ ^30^. 

The survey “Nursing Profile in Brazil,” conducted in 2016 by the Federal Council of Nursing (COFEN) and the Regional Councils of Nursing [COREN(s)], in partnership with the Oswaldo Cruz Foundation (FIOCRUZ) and published in 2017 found similar data to the present study regarding embarrassment and/or violence in the course of work. In the survey, 28.7% of professionals said they had been exposed to violence during their work. In the present study, 24.6% of the participants claimed to have suffered some kind of embarrassment and/or violence at work. Similar data were also found in a study conducted in São Paulo^31^, where 32.8% of the participants reported having experienced, at least, one episode of violence in the year prior to the survey. 

Professionals exposed to violence at work develop more symptoms of psychopathological distress than those not exposed^32^. This violence, most often committed by users, is pointed out by the workers themselves as impacting their health and is associated with Minor Mental Disorders, Burnout Syndrome, and reduced well-being at work^30^ ^,^ ^33^. Besides compromising the mental and physical health of the professionals, these acts of violence reflect negatively on the work processes of the health services^34^.

Most participants in the present survey, 391 (73.5%), stated that they did not receive psychological/emotional support from the institution where they work or study and this lack of support was also associated with all domains of the SAS-40 scale. This is an interesting finding, since although the State of São Paulo pioneered the detection of SARS-CoV-2 in Brazil^2^, did not, simultaneously, pay attention to mental suffering from COVID-19 in various population groups. 

 In this path, the United Nations highlights that the preservation of the mental health of health workers is a fundamental element in the actions of pandemic preparedness, response, and recovery instigated by COVID-19^35^. And, those on the front lines of care for patients with COVID-19 may develop mental disorders and other mental health symptoms^4^. 

Thus, it is of utmost importance that health services aim to guarantee biosafety, protection, organization and appropriate working conditions for all professionals, regardless of their category or institutional relationship. Given that the impacts on mental health, derived from increased levels of stress during the epidemic, can impair the attention and decision-making of workers, which impacts not only the management of actions against COVID-19 but also has an effect on their well-being after the epidemic period^9^. 

By revealing an overview of the physical and psychological burden on nursing professionals working in the most populated region of the country, which has the largest number of nursing workers and the highest technological density for the care of patients with COVID-19, this study supports the debate about the health needs and the conditions for facing the challenges imposed by the pandemic today and in the future for this group of workers. 

As limitations of this study that interfere with the generalization of the results, we highlight the time frame and the non-probabilistic sample. Even with the efforts to access more nursing professionals from the other states of the Southeast Region, we could not get a sample similar or close to that of the State of São Paulo. Therefore, further studies are suggested regarding the psychopathological symptoms and the work situation of nursing professionals in the context of the COVID-19 pandemic to expand the debate in the area of occupational health.

## Conclusion

In this study, the factors that were most related to psychopathological symptoms in the group working in the Southeast Region were: age group; weekly workload; and suffering embarrassment and/or violence in the course of work, since the pandemic state was declared by COVID-19. 

The aforementioned results pointed out the importance of increasingly earlier psychological interventions during and after the pandemic. Thus, it is suggested the creation of guidelines for the reception, adherence, and follow-up of nursing professionals through institutional proposals of support for emotional demands. 

## References

[1] Buss LF, Prete CA, Abrahim CM, Mendrone A, Salomon T, Almeida-Neto C (2021). Three-quarters attack rate of SARS-CoV-2 in the Brazilian Amazon during a largely unmitigated epidemic. Science.

[2] Cândido DS, Claro IM, De Jesus JG, Souza WM, Moreira FR, Dellicour S (2020). Evolution and epidemic spread of SARS-CoV-2 in Brazil. Science.

[3] Croda J, Oliveira WK, Frutuoso RL, Mandetta LH, Baia-da-Silva DC, Brito-Sousa JD (2020). COVID-19 in Brazil: Advantages of a socialized unified health system and preparation contain cases. Rev Soc Bras Med Trop.

[4] MoreiraWC Sousa AR, Nóbrega MPSS (2020). Mental illness in the general population and health professionals during COVID-19: a scoping review. Texto Contexto Enferm.

[5] Barroso BIDL, Souza MBCAD, Bregalda MM, Lancman S, Costa VBBD (2020). Worker health in COVID-19 times: reflections on health, safety, and occupational therapy. Cad Bras Ter Ocup.

[6] Conselho Federal de Enfermagem (BR) (2020). Profissionais infectados com COVID-19 informado pelos enfermeiros responsáveis técnicos/coordenadores.

[7] Sampaio F, Sequeira C, Teixeira L (2020). Nurses' Mental Health During the Covid-19 Outbreak. J Occup Environ Med.

[8] Góes FGB, Silva ACSSD, Santos ASTD, Pereira-Ávila FMV, Silva LJD, Silva LFD (2020). Challenges faced by pediatric nursing workers in the face of the COVID-19 pandemic. Rev. Latino-Am. Enfermagem.

[9] Kang L, Li Y, Hu S, Chen M, Yang C, Yang BX (2020). The mental health of medical workers in Wuhan, China dealing with the 2019 novel coronavirus. Lancet Psychiatry.

[10] Honein MA, Christie A, Rose DA, Brooks JT, Meaney-Delman D, Cohn A (2020). Summary of guidance for public health strategies to address high levels of community transmission of SARS-CoV-2 and related deaths, December 2020. Morb Mortal Wkly Rep.

[11] Xiao C (2020). A novel approach of consultation on 2019 novel coronavirus (COVID-19)-related psychological and mental problems: Structured letter therapy. Psychiatry Investig.

[12] Ornell F, Schuch JB, Sordi AO, Henrique F, Kesseler P (2020). Pandemic fear and COVID-19: mental health burden and strategies. Braz J Psychiatry.

[13] Shigemura J, Ursano RJ, Morganstein JC, Kurosawa M, Benedek DM (2020). Public responses to the novel 2019 coronavirus (2019-nCoV) in Japan: Mental health consequences and target populations. Psychiatry Clin Neurosci.

[14] Cheng A, Kessler D, Mackinnon R, Chang TP, Nadkarni VM, Hunt EA (2016). Reporting Guidelines for Health Care Simulation Research. Simul Healthc J Soc Simul Healthc.

[15] Ministério da Saúde (2020). Portaria 580 de 27 de março de 2020. Dispõe sobre a Ação Estratégica "O Brasil Conta Comigo - Residentes na área de Saúde", para o enfrentamento à pandemia do coronavírus (COVID-19). Diário Oficial da União.

[16] Derogatis LR (1994). Symptom Checklist-90-R (SCL-90-R) Administration, Scoring, and Procedures Manual.

[17] Laloni DT (2001). Escala de Avaliação de Sintomas-90-R SCL-90-R: adaptação, precisão e validade.

[18] Yoshida EMP, Silva FRCS (2007). Escala de Avaliação de Sintomas-40 (EAS-40): Validade e precisão em amostra não clínica. Psicol Esc Educ.

[19] Liu J, Liao X, Qian S, Yuan J, Wang F, Liu Y (2020). Community transmission of severe acute respiratory syndrome coronavirus 2, Shenzhen, China, 2020. Emerg Infect Dis.

[20] Wang C, Pan R, Wan X, Tan Y, Xu L, Ho CS (2020). Immediate Psychologial Response and Associated Factors during the Initial Stage of the 2019 Coronavirus Disease (COVID-19) Epidemic among the General Population in China. Int J Environ Res Public Health.

[21] Li H, Zhang Y, Wang H, Liang J, Zhou Y, Huang Y (2020). The relationship between symptoms of anxiety and somatic symptoms in health professionals during the coronavirus disease 2019 Pandemic. Neuropsychiatr Dis Treat.

[22] Su TP, Lien TC, Yang CY, Su YL, Wang JH, Tsai SL (2007). Prevalence of psychiatric morbidity and psychological adaptation of the nurses in a structured SARS caring unit during outbreak: a prospective and periodic assessment study in Taiwan. J Psychiatr Res.

[23] Lai J, Ma S, Wang Y, Cai Z, Hu J, Wei N (2020). Factors Associated with Mental Health Outcomes Among Health Care Workers Exposed to Coronavirus Disease 2019. JAMA.

[24] Fehn A, Nunes L, Aguillar A, Dal Poz M (2020). Vulnerabilidade e déficit de profissionais de saúde no enfrentamento da COVID-19. Nota técnica 10.

[25] Vedovato TG, Andrade CB, Santos DL, Bitencourt SM, Almeida LPD, Sampaio JFDS (2021). Health workers and COVID-19: flailing working conditions?. Rev Bras Saúde Ocup.

[26] Sousa KHJF, Zeitoune RCG, Portela LF, Tracera GMP, Moraes KG, Figueiró RFS (2020). Factors related to the risk of illness of nursing staff at work in a psychiatric institution. Rev. Latino-Am. Enfermagem.

[27] Lima EP, Assunção AA (2011). Prevalence and factors associated with Posttraumatic Stress Disorder (PTSD) in emergency workers: a systematic literature review. Rev Bras Epidemiol.

[28] Zhang WR, Wang K, Yin L, Zhao WF, Xue Q, Peng M (2020). Mental Health and Psychosocial Problems of Medical Health Workers during the COVID-19 Epidemic in China. Psychother Psychosom.

[29] Silva IV, Aquino EML, Pinto ICM (2014). Workplace violence in the healthcare sector: the experience of State health employees in Bahia State, Brazil. Cad Saúde Pública.

[30] Dal Pai D, Sturbelle ICS, Santos C, Tavares JP, Lautert L (2018). Physical and psychological violence in the workplace of healthcare professionals. Texto Contexto Enferm.

[31] Da Silva ATC, Peres MFT, Lopes CS, Schraiber LB, Susser E, Menezes PR (2015). Violence at work and depressive symptoms in primary health care teams: a cross-sectional study in Brazil. Soc Psychiatry Psychiatr Epidemiol.

[32] Jaradat Y, Nilsen MB, Kristensen P, Nijem K, Bjertness E, Stigum H (2016). Workplace aggression, psychological distress, and job satisfaction among Palestinian nurses: A cross-sectional study. Appl Nurs Res.

[33] Bernaldo-de-Quirós M, Labrador FJ, Piccino AT, Gómes MJC (2014). Workplace violence in prehospital emergency care: A systematic review and outlines of psychological intervention: Second prize of the 20th "Rafael Burgaleta" Applied Psychology Awards 2013. Clin Salud.

[34] Pousa PCP, Lucca SR (2021). Psychosocial factors in nursing work and occupational risks: a systematic review. Rev Bras Enferm.

[35] Fundação Oswaldo Cruz (2020). Saúde Mental e Atenção Psicossocial na Pandemia COVID-19. Recomendações para Gestores.

